# Can *Plasmodium*’s tricks for enhancing its transmission be turned against the parasite? New hopes for vector control

**DOI:** 10.1080/20477724.2019.1703398

**Published:** 2020-01-07

**Authors:** S. Noushin Emami, Melika Hajkazemian, Raimondas Mozūraitis

**Affiliations:** a Department of Molecular Biosciences, Wenner-Gren Institute, Stockholm University, Stockholm, Sweden; b Department of Zoology, Stockholm University, Stockholm, Sweden; c Laboratory of Chemical and Behavioral Ecology, Institute of Ecology, Nature Research Centre, Vilnius, Lithuania

**Keywords:** *Plasmodium* induced volatile organic compounds (VOCs), mosquito, host-seeking, phago-stimulation, vector control

## Abstract

Approximately 120 years ago the link between mosquito and the malaria transmission was discovered. However, even today it remains an open question whether the parasite is able to direct the blood-seeking and feeding behavior of its mosquito vector to maximize the probability of transmission. If the parasite has this ability, could it occur only through the alteration of the vertebrate host’s volatile organic compounds (VOCs) and/or the parasite alteration of the behavior of the infected vector in a manner that favors its transmission? Although some recent empirical evidence supports the hypothesis regarding the parasite ability in alteration of the vertebrate host’s VOCs, the role of parasite alteration and behavioral differences between infected and uninfected female mosquitoes toward infected and uninfected hosts has not yet been considered in the implementation of control measures. This review will discuss the current evidence, which shows 1. *Plasmodium* can direct uninfected mosquito blood-seeking and feeding behavior via alteration of vertebrate-host odor profiles and production of phagostimulants and 2. *Plasmodium* also manipulates its vector during the sporogony cycle to increase transmission. Briefly, we also consider the next generation of methods for moving the empirical laboratory evidence to potential application in future integrated malaria control programs.

## Introduction

**Figure 1.**
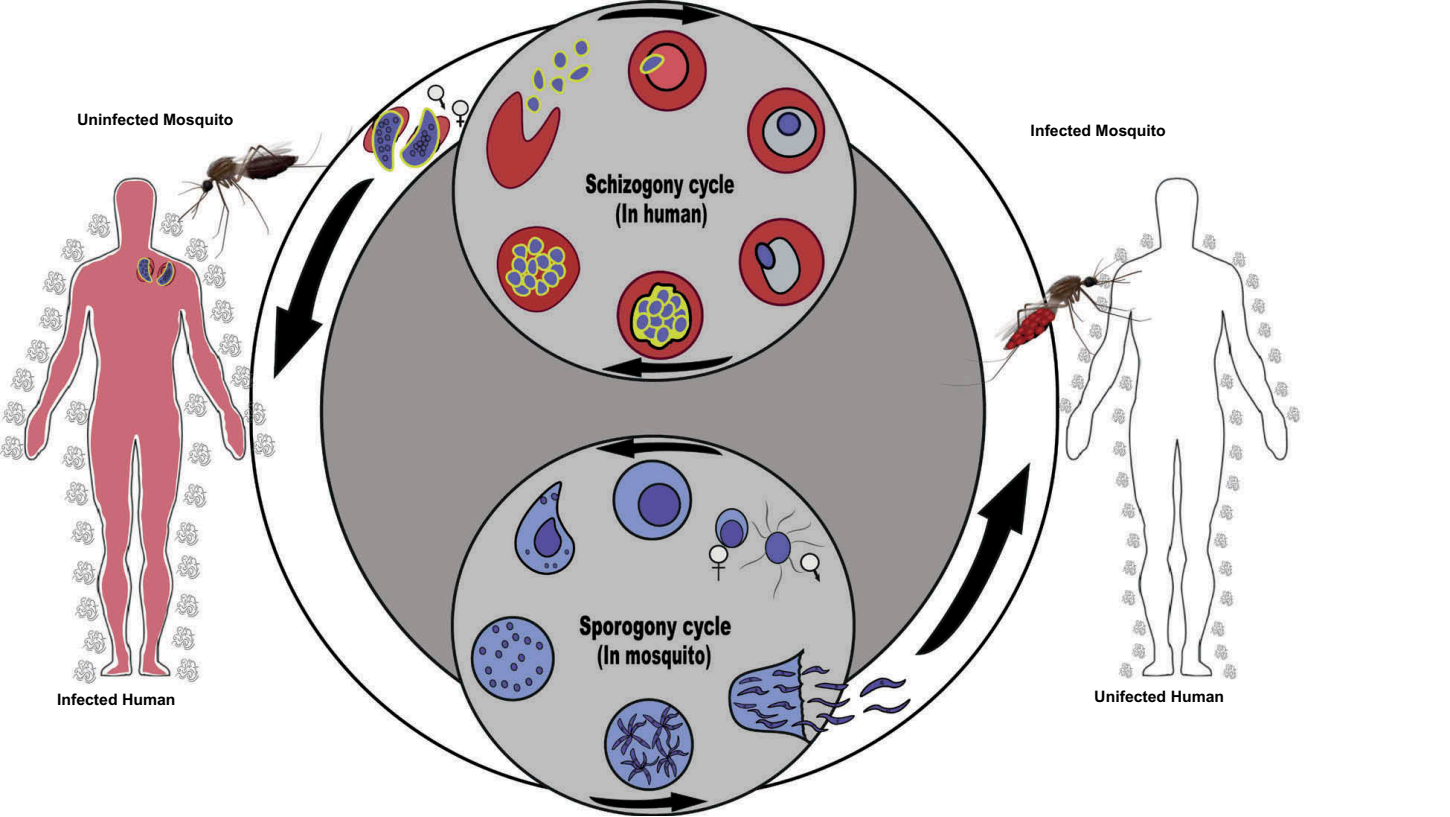
*Plasmodium falciparum* transmission cycle. The human malaria transmission cycle, including the first bite of an uninfected mosquito (top left) on a gametocyte carrier (infective human: schizogony cycle). Mature gametocytes are taken up by female mosquitoes, initiating the mosquito developmental stages of the parasites (sporogony cycle), and during this stage the parasite follows replication stages and the mosquito becomes infected during ~7–10 days and infective ~11–14 days. When an infective mosquito bites an uninfected human host (top right), it can transmit the infectious stage (sporozoites) to a second human host and malaria transmission cycle continues.

A key requirement for transmission of malaria parasites is the uptake of an infected blood meal by a mosquito which initiates the parasite transmission cycle (Figure 1). The rate of transmission, as shown by Ross-MacDonald equation, is exponentially related to the human biting rate, which is the proportion of the mosquitoes that feed on humans each day [1,2]. The host seeking and biting rate of *Anopheles* mosquitoes on an individual human host are suggested to be correlated with host skin microbial composition and, recent work, also identified malaria parasite-associated cues [3,4].

Mosquitoes’ unique bionomics guarantee an efficient vector–host interaction, favorable for malaria transmission. Mosquitoes use several senses to locate and choose a host, predominantly olfaction [5,6]. The semiochemical basis of human host seeking has been studied during past years [7–9], allowing us to further characterize the basis of this important behavior. The best-known cue for activation and attraction is carbon dioxide (CO_2_), which is emitted by vertebrates through red blood cells, breath and skin, and indicates the presence of a potential host [6,10,11]. Mosquitoes also respond to the temperature of mammals and birds and the faster transport of odor by the heat convention current, resulting from metabolic activity [12,13]. Body heat and mass have a direct effect on the dispersal of chemicals, thus influencing mosquito [*An. atroparvus* and *An. gambiae* s.s.] attraction to the host behavior [14]. Once mosquitoes detect, land, and start to feed on a suitable host the volume and speed at which blood is taken varies. This complex behavior is dependent on feeding stimulation, olfactory apparatus, host defense, and neurophysiological pathways connected to the mosquito’s mouthparts which consequently boost the basic reproduction rate of pathogen transmission as well [5,15,16]. The avidity of the vector for a blood meal is measured as persistence (time spent feeding) and the number of times a mosquito attempts to find blood by inserting its proboscis (probing). Increased probing can enhance the chance of malaria parasite sporozoite transmission to the host blood vessels. It also risks being killed by the host defense or picking up of increased dose of insecticide. The volume of blood taken, and the speed of feeding has a direct effect on the likelihood of a mosquito becoming infected and subsequently transmitting the parasite [17]. Larger blood meals increase the chances of taking up infectious gametocytes and therefore infection and faster feeding rates are correlated with higher survival of mosquitoes due to host bite avoidance behavior. Whatever the mechanisms responsible for blood seeking/feeding behavior, malaria parasite seems to modify them to increase its transmission.

Infamous pathogen vectors such as other anthropophilic anopheles such as *An. funestus* and *An gambiae* tend not to feed on sugar when human blood are available [18]. Carbohydrate availability is important for the distance a mosquito is able to fly (mosquito flight range). Mosquitoes with low carbohydrate reserves have limited flight ability [19]. Mosquitoes can also utilize part of the blood meal to produce the energy needed for flight; the remainder of the digestion products are converted to glycogen and triacylglycerol. Approximately 30% of the energy value of the blood meal is used for energy production in *An. gambiae* [20]. Blood-fed *An. gambiae* can fly further than those fed on sugar alone [14,20], but *An. atroparvus* can fly further with sugar-only – this species does not seem to be able to use the lipids from blood efficiently for glucose production [14,20]. If extra sugar meal makes infectious mosquitoes capable of longer flight, they have an increased chance of finding a host, and therefore may have an increased likelihood of being a major risk for pathogen transmission.

Here, the direct effect of *Plasmodium* on (1) uninfected mosquito blood-seeking and feeding behavior, and (2) manipulation of its vector during sporogony cycle will be discussed in detail in the following sections (3). We will also summarize whether parasites are virulent to their vectors based on published literature. Finally, the next generation of methods for moving the empirical laboratory evidence to potential application in future integrated malaria control programs will be indicated.

## Parasite–vector interplay for the transmission success

The complex biology of the *Plasmodium* parasite includes sexual development in their definitive hosts, particularly mosquito species within the genus *Anopheles*. The parasite cycle starts with the desire of mosquitoes to uptake blood. The mosquito must ingest male and female sexual forms of the parasite in order to become infected and capable of subsequently infecting another human host. Vector-borne parasites may also alter the vector’s behavior to increase their transmission ability [21]. The mechanisms by which the presence of the malaria parasite could influence malaria transmission by altering the senses of the vectors are discussed below.

All malaria parasites require two hosts, the vertebrate host where schizogony occurs within the erythrocytes (human blood stages of parasite), and the mosquito vector where sporogony (mosquito stages of parasite) occurs in the mosquito midgut wall [22]. In the vertebrate host, an infection is initiated when sporozoites are injected with the saliva of an infected mosquito, and parasites replicate asexually, first in the liver, and then in the erythrocytes (reviewed in [23]). Whilst the majority of parasites invading erythrocytes are destined to continue through the asexual cycle, a small fraction can develop into gametocytes, the first sexual stage of the life cycle. In *Plasmodium falciparum*, the presence of gametocytes in the peripheral blood appears 7–15 days after the initial asexual wave which is long compared to the other human malaria species 1–3 days. The ratio of gametocytes to asexual stages in *P. falciparum* is usually found at very low levels [24]. Only people carrying gametocytes can be infectious to mosquitoes. Recent studies showed that microscopy is insufficiently sensitive to detect low densities of asexual parasites and gametocyte [25,26]. In most endemic settings, a small proportion of infecting parasites are gametocytes [27].

Mosquitoes become infected after biting malaria-infected people carrying the gametocytes; there is a complex period of sexual reproduction and development within the mosquito that results in sporozoite infection in the salivary glands, infective mosquitoes [22]. Human malaria parasites alter the probing and persistence behavior of mosquitoes, resulting in increased transmission [28]. There is also evidence that parasites and their metabolites affect both mosquito and human host physiology and behavior to increase the probability of transmission [11,21,29–33]. In rodent and bird malaria model systems, infected hosts have been found to be more attractive to mosquitoes [30,34]. The same has been observed for humans infected with both *Plasmodium falciparum* [35] and *Plasmodium vivax* [36]. It has also been reported that salivary gland malaria-infected mosquitoes show increased attraction to human odor [37].

## *1 – Plasmodium* direct the host-seeking mosquitoes toward infected hosts (manipulation beyond the host)

Does transmission begin with an ancient chemical coding? Mosquitoes favor malaria-infected individuals, especially hosts carrying gametocytes or parasite signaling metabolites, more frequently than uninfected individuals [4,11,35,36]. This phenomenon can be influenced by two routes, direct emission of volatile organic compounds and/or skin microbial composition (reviewed in [4]). Increased vector attraction to the infected vertebrate host would provide an evolutionary advantage to the parasite as it increases host-vector contact, thus likely enhancing the chances of transmission [38].

Emami *et al*. [11] recently showed enhanced mosquito preference for blood containing a *P. falciparum* metabolite, (*E*)-4-hydroxy-3-methyl-but-2-enyl pyrophosphate (HMBPP) over parasite-free blood due to enhanced release of certain volatiles. In the field, western Kenya, children harboring gametocytes of *P. falciparum* attracted about twice as many mosquitoes compared to naturally infect with asexual stage and uninfected children [35]. Increased vertebrate-host attraction during non-transmissible stages of the parasite would be disadvantageous to the malaria parasite since vertebrates frequently swat mosquitoes; hence, there is a higher risk of vector mortality. However, a recent study on attractiveness of *P. vivax* gametocyte carriers which has been grouped the patients into fever and non-fever categories. They showed a higher attractiveness to mosquitoes only in the fever group of gametocyte carriers, indicating a synergistic effect of temperature and gametocytes in the host attractiveness to *Anopheles darlingi* [36]. There is a need for high-quality studies of the interactions and differences in clinical symptoms, associated pathological factors and gametocytaemia in malaria due to *P. vivax* and *P. falciparum*. In summary, we believe these studies usher in a new era in the investigation of the host–pathogen interaction with exciting opportunities to counteract the malarial scourge on human lives.

## *Plasmodium* induced volatile organic compounds (VOCs) and mosquito olfaction

The olfactory apparatus of mosquitoes comprises three specialized head appendages, the antennae, maxillary palp and labella, that carry specialized sensory hairs called sensilla that typically contain two to three olfactory sensory neurons (OSNs). At the molecular level, OSNs are thought to express unique types of chemosensory receptors on dendritic membrane to detect a wide array of environmental odorants, reviewed in [39]. Like in other insects, the genome of *An. gambiae* and *Ae. aegypti* encodes three classes of chemosensory receptors. These chemoreceptors include odorant receptors (*Ors*), ionotropic receptors (*Irs*) and gustatory receptors (*Grs*) that are devoted to detect a range of cues associated with mosquito host-seeking, feeding, oviposition and several other key behaviors that enhance their functional traits [40,41].

It is well documented that mosquito search behavior for hosts is triggered by olfactory perception of VOCs emitted from animals and humans [30,42,43]. *Plasmodium* parasites are able to increase the infected host attraction to vectors by manipulating host-VOC profiles [30,44]. A recent study demonstrated increases in the production of the heptanal, octanal, nonanal, (*E*)-2-octenal, (*E*)-2-decenal and 2-octanone by malaria parasite-infected Kenyan children. Increases were broadly associated with infections with high malaria parasite load, compared to patients having either low malaria parasite density or being parasite-free [44]. Kelly et al. [45] reported that *P. falciparum* infected erythrocytes released few VOCs under in vitro culture conditions. Amounts of the monoterpene, α-pinene, were higher in the samples containing erythrocytes infected by *P. falciparum* strain 3D7-MR4 at 2% parasitemia compared to those bearing uninfected erythrocytes as well as to the samples containing infected erythrocytes treated with 5 µM of fosmidomycin [45], an inhibitor of parasite isoprenoid biosynthesis by affecting the first enzyme of the MEP pathway, deoxyxylulose phosphate reductoisomerase [46, 47]. Identification of other four compounds, namely, 3,5-bis (1,1-dimethylethyl)-4-methyl-1H-pyrazole, 6-hydroxy-5,7-dimethoxy-naphtho[2,3-c]furan-1(3H)-one, 4,5,9,10-dehydro-isolongifolene and 8,9-dehydro-9-formyl cycloisolongifolene is putative, due to insufficient match factors of mass spectra obtained from analytes and those available in electronic libraries as well as the absence of standards during identification [45]. Recently, Emami and colleagues [11] showed that a *P. falciparum*-derived compound, HMBPP, significantly enhances the release of CO_2_ by 16% in the headspace of treated erythrocytes compared to untreated cells. HMBPP addition also increased the release of three monoterpenes, α- and ß-pinene and limonene by 1.2-to-1.6 fold as well as three aldehydes octanal, nonanal and decanal by 1.7-to-5.2 fold. A mixture of all seven components was essential to trigger the host-seeking behavior of *An. gambiae* s.s. under laboratory conditions. These findings suggest the mechanism which underpins the *Plasmodium*’s requirement to manipulate their mosquito hosts in order to elevate the odds of infection to a degree sufficient for success at sustained transmission. These elements of the chemical signaling belonged to three main categories of volatiles, CO_2_, aldehydes and terpenes and appear highly evolved between these parasites and their definitive hosts, suggesting a powerful selection and survival advantage linked to it [11].

A study using mice malaria-mosquito system revealed that mice at the chronic stage harboring relatively high levels of *Plasmodium chabaudi* (clone AS) gametocytes emitted significantly larger amounts of N,N-dibutylformamide, tridecane, 2-pyrrolidone, 2-hexanone, 2-methyl-butanoic acid, 2-phenyl ethanol and two unidentified compounds. In addition, amounts of two acids, namely 3-methyl butanoic and hexanoic acids tend to differ from the emissions of infected versus uninfected mice. It is interesting to note that in the 3 months after initial infection, volatile collections showed a decrease in overall volatile emissions from infected mice during the early, acute stage of infection compared to healthy controls as well as periodic cycles of elevated volatile emissions which corresponded to increase attractiveness of mice to mosquitoes [30].

**Figure 2.**
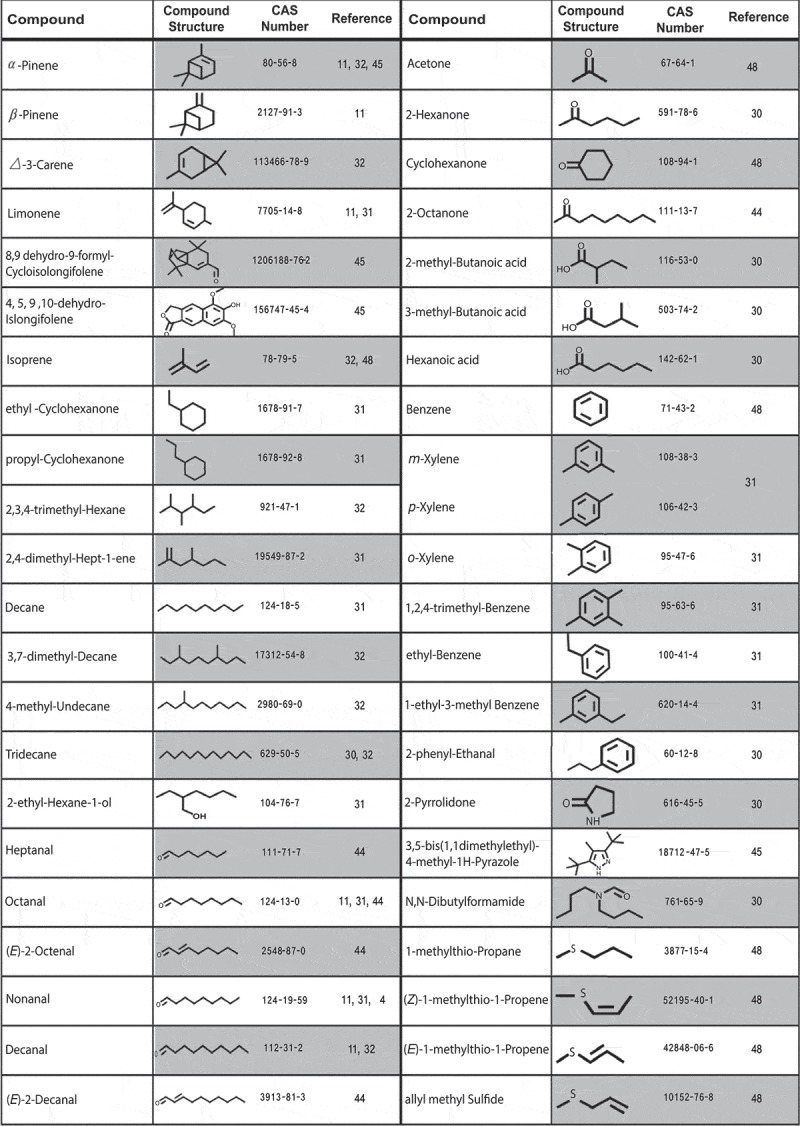
*Plasmodium* induced volatile organic compounds (VOCs). *Plasmodium* associated compounds implicated in host responses (*in vitro* or *in vivo* studies). Chemical structures of volatile compounds have been shown. CAS indicates Chemical Abstracts Service which provides unique numerical identifier to every chemical substance described in the open scientific literature.

Of note, there are studies which followed infected individuals by detecting and quantifying parasite manipulated VOCs. Odors present in breath of symptomatic malaria-infected children (80% of pediatric patients with fever) and healthy individuals as a control were sampled in a medical facility. The samples were collected on absorbent resins in sorbent tubes which were later transported to the laboratory. The breath composition was analyzed and chromatographic patterns obtained from infected and healthy individuals were compared aiming to find malaria-associated volatile chemical markers. Data analyses revealed a cumulative abundance metric with a set of six VOCs, 4-methyl-undecane, 3,7-dimethyl-decane, 2,3,4-trimethyl-hexane, nonanal, tridecane and isoprene [32]. In addition, it was found that infection correlates with significantly higher breath levels of two mosquito-attractant terpenes, α-pinene and 3-carene. This work was conducted in Malawi [32]. However, another breathprint study of infected *P. falciparum* volunteers reported other compounds including isoprene, acetone, benzene, cyclohexanone, allyl methyl sulfide, 1-methylthio-propane, (*Z*)-1-methylthio-1-propene, and (*E*)-1-methylthio-1-propene [48]. The major difference in these studies was that in the experimentally induced blood-stage malaria model, patients had very low parasitaemias (i.e. the percentage of RBCs that contained parasite to uninfected RBCs), being on the order of 100,000-fold lower than in the Malawian patients (parasitaemias ~2%). It is also plausible that Malawian patients were likely to harbor mature gametocytes which are the transmissible form of blood stage of parasite (human infection) to mosquitoes. A study of Kenyan patients also reported that malaria patients who carried the gametocyte stage of *P. falciparum* parasite were highly attractive to *An. gambiae* vectors [49]. An extensive collections of skin volatiles from primary-school children with high rates of malaria infection in western Kenya revealed consistent effects of malaria infection on human volatile profiles, as well as significant divergence in the effects of symptomatic versus asymptomatic infections. Ten volatiles including ethyl-cyclohexane, ethyl-benzene, m-xylene or p-xylene, o-xylene, propylcyclohexane, 1-ethyl-3-methylbenzene, decane, nonanal, and two unidentified compounds were released at significantly higher amounts in asymptomatic compared to uninfected individuals. In symptomatic individuals, amounts of six compounds, namely, 2,4-dimethylhept-1-ene, 1-ethyl-3-methylbenzene, 1,2,4-trimethylbenzene, decane, s(-)-limonene, and 2-ethylhexan-1-ol were significantly higher compared to those in uninfected individuals (Figure 2) [31]. These studies highlighted that the mature gametocyte (sexual stage of the parasite in human blood) could have been the key contributor to the VOCs [50], chemical codes which communicate between mosquito and parasite to enhance the efficiency of parasite transmission (Figure 2). Ultimately, what will be happened to parasite transmission success, once the decoding chemical information (VOCs) of parasite go straight to what the vector control strategies are conveying? We highlight key knowledge gaps that need to be addressed for the successful development of strategies that interrupt malaria transmission.

## *Plasmodium* metabolite and mosquito gustation

Based on studies in human and other primates, two putative *P. falciparum* derived elicitors, glycosylphosphatidyl-inositols (GPIs) and hemozoin (Hz), have been investigated previously in mosquito vectors. GPIs function as universal cell membrane anchors for proteins and are the most common carbohydrate modification in *Plasmodium* [51]. The GPI anchors are necessary for parasite survival and they must be synthesized *de novo* during intraerythrocytic stages [52]. *P. falciparum* GPIs mediate many of the symptoms associated with severe human malaria pathophysiology. It also influences the mosquito immune system and suppressed fecundity strongly and the egg viability of *An. gambiae* (reviewed in [53]). Provision of PfGPIs in the blood meal induces nitric oxide synthase (NOS) through extracellular signal-regulated kinase (ERK)/protein kinase B (Akt) signaling in the midgut of *An. stephensi* [54]. *P. falciparum* GPIs also induce several AMP genes and decrease fecundity in *An. gambiae* mosquitoes [55]. *Plasmodium* Hz is a brownish pigment generated during digestion of hemoglobin with host red blood cells [56]. *P. falciparum* Hz can also induce NOS and stimulate *An. gambiae* and *An. stephensi* cell lines as well as in *An. stephensi* midgut tissues [57].

Isoprenoids are widespread molecules and are necessary for all living organisms [58]. These molecules are involved in a vast spectrum of metabolic processes and serve as building blocks in the synthesis of various compounds such as cholesterol, steroid hormones and vitamins [59]. Animals, fungi and a few bacteria produce isoprenoids through a biosynthetic route called mevalonate pathway. By contrast, eubacteria, plastid-containing eukaryotes and most bacteria use an alternate metabolic route, the non-mevalonate or methylerythritol phosphate (MEP) pathway. Plants use both pathways, the chloroplast-localized MEP pathway that is used for biosynthesis of the terpene volatiles that contributs their characteristic flavors and fragrances [60]. MEP pathway is used by parasitic apicomplaxan protozoa, including *Plasmodium* (reviewed in [61]). The MEP pathway is one of the recognizable pathways in malaria parasite apicoplast and this pathway might have evolved due to its lower energy consumption (reviewed in [45]). Due to its non-host specificity, biochemical reactions of MEP pathway have been favored as a highlighted target for novel antiparasitic drugs in human host. For example, fosmidomycin and its derivative, FR-900098 have an antibiotic activity that targets DOXP reductoisomerase and inhibits the growth of asexual stage of malaria parasite [62]. Parasites lost their apicoplast after non-antifolate antibiotic treatments such as doxycycline. Interestingly, parasite growth (asexual stage) is rescued upon simultaneous supplementation with the central isoprenoid precursor, isopentenyl pyrophosphate (IPP) [63].

Emami et al., 2017 recently published a compelling piece of evidence that the presence of one of the malaria parasite metabolites, called (*E*)-4-hydroxy-3-methyl-but-2-enyl pyrophosphate (HMBPP) in a blood meal, or in a meal of serum or physiological saline, is sufficient to stimulate 80–100% of the *An. gambiae* s.l. to engorge. This metabolite doubles the number of mosquitoes that will engorge on a blood meal alone [11]. Previously, it has been shown that all blood-sucking insects have one phagostimulant in common, adenosine triphosphate (ATP) identified in mosquitoes [64,65]; tsetse flies [66]; tabanids [67]; simulids [68]; fleas [69] and rhodnius [70]. With regards to malaria cycle, there is some evidence that *Plasmodium* metabolites, possibly other protozoans metabolites, and conserved signaling pathways might manipulate host/vector metabolism and feeding behavior, for offering a unique opportunity in the animal kingdom that intersect across more than 600 million years of evolution (reviewed in [71]).

## *2 – Plasmodium* manipulates its vector during the sporogony cycle (manipulation within the host)

Parasite development in the mosquito can also affect mosquito behavior. Mosquitoes are more avid to bite a host when malaria parasites have developed into transmissible sporozoites in the salivary glands. Laboratory evidence showed that feeding persistence of female *An. stephensi* mosquitoes depends on the presence and development stage of the parasite (*Plasmodium yoelii nigeriensis*). In 10-min trials, it was shown that 33% of uninfected, 53% of those harboring oocysts and only 20% of those infected with sporozoite mosquitoes, respectively, gave up feeding on human host [72]. It seems that the feeding behavior of mosquitoes is not only mediated by the presence of parasite but also it is sensitive to the developmental stage of parasite. Mosquito are more eager to bite the host when malaria parasite has developed into transmissible sporozoites. Also, field study showed that sporozoite-infected mosquitoes took larger blood meals than uninfected mosquitoes and 82% of infected mosquitoes had engorged fully. In addition, individual mosquitoes carrying sporozoite were more likely to bite several people per night [73]. Mosquitoes infected with sporozoites of *Plasmodium gallinaceum* needed a larger blood meal to inhibit host-seeking behavior than those not infected [74]. For example, *An. gambiae* and *Anopheles punctulatus* infected by *P. falciparum* and *P. vivax* at sporozoite stage attempted to take more blood meals than uninfected mosquitoes, a behavior which could increase the rate of parasite transmission by increasing their biting rate [17,73].

## *Plasmodium* and Anophelines interplay in response to the interventions against them

Complex biology of the *plasmodium* parasite, differences in vector capacity as well as biological and ecological features of vector mosquitoes requires integrated approach to malaria control [75]. To achieve malaria reduction and elimination goals, the World Health Organization (WHO) has recommended wide-scale actions falling into three categories: (i) efficient diagnosis and treatment of disease; (ii) quality-assured vector control; and (iii) preventive treatments [76].

The clinical symptoms of malaria are nonspecific. Consequently, parasite-specific laboratory tests must be carried out to confirm the infection [77]. Currently, microscopic examination of stained blood films and rapid diagnostic tests (RDTs) detecting parasite antigens are the most common tests [78]. In 2016 alone, an estimated 312 million RDTs were delivered globally, of which 269 million were in Africa [79]. Most of RDTs tests rely on the detection of the particular pathogen’s proteins. For example, in the case of the malaria, *P. falciparum* histidine-rich protein HRP2 is selected. The hrp2 gene, however, is dispensable, and parasite strains that have deleted this gene and escape RDT detection are on the rise. In a recent modeling study, false-negative rates with HRP2-based tests were predicted to attain 20% in many parts of Africa by 2030 [80]. Nucleic acid amplification tests (NAAT) are superior for detecting mixed infections, cases with low parasitemia. Nucleic acid amplification tests also use commonly for the identification of species when the parasite morphology is inadequate for microscopic identification. Although nucleic acid amplification tests resolve discrepant results compare to other methodologies, however, the method is expensive and highly-complex [78]. Serologic tests are used in cases where parasites are not seen on peripheral blood smears, for screening donors and in some other occasions. However, the tests cannot differentiate between past and current infections [78]. There are also different lines of research for identifying malaria parasites in blood smears using new methods such as a Focal Plane Array (FPA), Fourier transform infrared imaging spectroscopy. This technique, in combination with hyper-spectral processing, enables imaging and diagnosis of early stage malaria parasites at the single cell level in a blood smear [81]. The next one is magneto-optic technology which is also used for early stage malaria diagnosis in human based on the detection of the malaria pigment, hemozoin [82]. These methods will be challenging; however, recent advances suggest that they may be a possibility in the foreseeable future.

The accumulating data that pathogens manipulate host odor profiles to influence vector behavior reveal a possibility for developing new non-interventional, volatile-based malaria detection methods that have to be reliable, user-friendly, and affordably for the screening of human populations. Number of studies dealing with VOCs collection from human skin, breath, or blood showed volatile signature of parasite presence. Certain volatiles have been repeatedly found as indicators of the presence of malaria parasite, for example, octanal, nonanal, decanal, isoprene, tridecane, α-pinene and limonene (Figure 2). However, a single study has been reported that the amount of these volatiles can significantly differ with respect to the presence/absence of parasite as well as the presence of various parasite stages with regard to large structural diversity (Figure 2). Moreover, so far no parasite-specific compound has been identified, except in the study that is carried out by Kelly *et al*. [47] where identifications remain putative and have to be confirmed. The absence of parasite-specific volatiles is in accordance with the deceptive chemical signal hypothesis that predicts parasite manipulates existing host-cues rather than produces novel signals resulting in increased attractiveness of infected vertebrate hosts [83,84]. It is much more challenging to build a model that classifies samples based on quantitative rather than the qualitative difference of diagnostic compounds. The next step will be to determine whether quantitative differences of diagnostic volatiles between healthy and different stage malaria bearing individuals are large enough with respect to genetic variation among individuals, geographical regions, and diet, as well as the presence of other pathogens and parasites.

Prevention of human contacts with malaria vectors relies almost wholly upon a few essential tools that greatly mitigate the harm done by malaria [79]. These tools include long-lasting insecticide-treated netting (LLINs), and indoor residual spraying of insecticides (IRS) where the latter method strongly contributed to malaria decline in sub-Saharan Africa [85]. Mosquitoes show an impressive ability to adapt to new environments; consequently, LLINs and IRS became vulnerable to the development of mosquito resistance and behavior changes. For example, mosquito resistance to the pyrethroid insecticides exclusively used in LLINs is emerging [86] and in the case of the many hundreds of millions of LLINs distributed for minimizing human contact with the night-feeding *anophelines*, selection for *anophelines* feeding earlier in the evening (before people retreat to their bed nets) or outdoors is occurring [87]. The rapid development of eco-friendly formulations of novel insecticides [88,89] and nanostructured materials [90] is a temporal solution to the vector control problem. Use of attractive toxic sugar baits (ATSB) had an environmental issue due to the lack of mosquito attraction specificity, and targeting beneficial insect including pollinators. Highly mosquito attractive synthetic odor blend mimicking human smell has been reported [91], which in turn could be further improved by imitating malaria-infected human dour. Malaria-infected human dour might specifically attract mosquitoes to toxic agent containing HMBPP, which stimulates toxin uptake. This lure and kill set up could be environment-friendly alternative to ATSB.

Eco-friendly mosquito control strategies such as sterile insect technique (SIT) and *Wolbachia* driven population suppression techniques as well as insects carrying a dominant lethal (RIDL) require mass production of mosquitoes [92,93]. Production of high-quality mosquitoes in mass amounts requires optimized breeding solutions, where phagostimulants are essential component ensuring sufficient engorgement rates of a meal. Adenosine triphosphate (ATP) is the most effective phagostimulant known for mosquitoes, however, ATP cannot be stored at room temperature and is relatively unstable in aqueous solutions [83]. Finding a replacement compound for ATP is therefore desirable. HMBPP is reported as a stable compound and is efficient as a phagostimulant at low concentrations. HMBPP might have the ability to replace ATP and reduce breeding cost in the mass production of mosquitoes.

The discovery of parasite metabolites (e.g. HMBPP) signaling points to a highly evolved ‘trick’ by the plasmodium spp., a chemical beacon guiding the anophelines not only to the rare host actually carrying parasites (as in most endemic areas globally, i.e., <2% prevalence), but also to sites on that host tissue bearing parasites for consumption [11,30,31]. These effects observed at miniscule concentrations (picomolar), suggesting an obligate dependency upon parasite signaling to improve the odds of mosquito infection. It is plausible that transmission in the wild may be unsustainable in the absence of the parasite chemical beacon. If so, switching off that beacon by any one of the many possible technological means may suffice to suppress the efficiency of anopheline infection to below sustainable levels. Those ways and means effectively represent a wide range of possible new molecular targets (in mosquitoes, parasites, and humans) for blocking malaria transmission among humans. At present, biochemical, genetic and crystallographic approaches with the MEP pathway enzymes are now starting to characterize the inhibition kinetics and identify which residues play a structural or catalytic role. However, there is still a gap in our knowledge regarding the transportation of these compounds out of malaria parasite-infected cells. The underlying mechanism by which HMBPP transports and mediates signaling has not been investigated. Current efforts should eventually contribute to an effective drug designed to fight against pathogens that show resistance to currently available agents.

A genetics-based population suppression and elimination approach is yet another strategy which might provide a better control to vector-borne diseases. In this system, the main problem is the means of driving a refractory construct quickly and efficiently through the vector mosquito population so that the population of susceptible mosquitoes will be replaced. Transposable elements (TEs) were one of the first gene drive systems to gain widespread attention for population replacement [94]. These elements are able to spread quickly through a population due to their ability to replicate within a host genome and hence to be inherited more frequently in the offspring’s genome. This increase in inheritance enables TEs to spread even in the presence of a fitness cost to the host [95]. One note, new techniques such as CRISPR-Cas9 gene drive targeting doublesex have also been reported a complete population suppression in caged *An. gambiae* mosquitoes [96]. Obviously, there is some doubts about the potential success of this strategy in malaria-endemic regions, where harboring a resistance allele will only be a piece of the complex puzzle of pathogen–vector interactions. Apart from the undeniable social and ethical issues associated with the release of genetically modified organisms [97], transgenic mosquitoes might still be considered as a realistic strategy for malaria control. The vigorous and technologically cutting-edge effort to produce a vaccine against malaria over the past four decades has yet to yield a useful product [98].

Finally, vector-borne disease caused by mosquitoes has been a threat to billions of human life around the globe. In such setting, the development of any new methods from novel mosquitoes lures to genetics-based population techniques that can control the transmission of the pathogens and/or population suppression and elimination ability is a promising approach. These new hopes could potentially reduce threats exerted by mosquitoes to humans. The work carried out so far has shown successful results in the laboratory can be the leading strategies in the future to control mosquito-borne diseases.

## Concluding remarks

The ability of parasites to manipulate the behavior of their hosts fascinates both scientists and non-scientists alike. Undoubtedly, the design and interpretation of investigations in this field is hampered by our inadequate understanding of the natural-based studies. Here we highlight the studies which show 1. *Plasmodium* can direct uninfected mosquito blood-seeking and feeding behavior and 2. *Plasmodium* also manipulates its vector during sporogony cycle to increase transmission. Ultimately, we discuss whether parasites are virulent to their vector-based on published literature. In our idea, the utility of studying the natural system of vector-host–parasite manipulations is to use them as potential avenues for further research into vector control strategies. We require new methods/tools to combat this scourge in order to avoid catastrophic loss of lives as conventional tools become increasingly ineffective or economically unsustainable. Although there is much in this age that is new, many of the current debates and arguments about malaria control are echoes of the past. History shows that gains made against malaria parasite are invariably eroded with great rapidity unless driven to local extinction. Experience from the past is a vital tool in the formulation of new malaria control. An understanding of the evolution and context of those challenges and innovative ideas can help us to direct the malaria control of today and the future.
